# TG‐43U1 parameterization of elongated RadioCoil P103d brachytherapy sources

**DOI:** 10.1120/jacmp.v8i3.2435

**Published:** 2007-07-17

**Authors:** Sharifeh A. Dini, Shahid B. Awan, Kai Dou, Ali S. Meigooni

**Affiliations:** ^1^ Department of Radiation Medicine University of Kentucky Chandler Medical Center Lexington Kentucky U.S.A.

**Keywords:** Brachytherapy, linear source, elongated sources, P103d, RadioCoil

## Abstract

Recently, to eliminate problems associated with seed type sources, RadioMed Corporation (Tyngsboro, MA) introduced RadioCoil P103d sources for interstitial prostate implants. The RadioCoil sources are available in integral lengths ranging from 1.0 cm to 6.0 cm. In this project, dosimetric characteristics of these sources were determined following the TG‐43U1 recommendations, with consideration of our recent publication on the evaluation of two‐dimensional anisotropy function for elongated brachytherapy sources. Dosimetric parameters of these sources were determined experimentally in Solid Water (Gammex RMI, Middleton, WI) and theoretically using Monte Carlo simulation in Solid Water and liquid water. Per the TG‐43U1 protocol, the consensus of these results would be used for their clinical applications.

PACS number: 87.53.Jw

## I. INTRODUCTION

Prostate brachytherapy with I125 and P103d sources is a treatment of choice for patients with early‐stage prostate cancer. Despite the enormous successes and improvements in interstitial brachytherapy, seed migration and embolization are two of the remaining risks associated with loose seed implants.^(^ ^1^ ^–^ ^5^ ^)^ Moreover, deviation of the seeds from the preplanned position is also linked with loose seed implants.^(^ ^6^ ^,^ ^7^ ^)^ To eliminate these problems, various pseudo‐linear (i.e. stranded) source models have been introduced. The stranded sources are fabricated by connecting a series of seeds in a linear fashion using a bio‐dissolvable material.^(3)^ Various investigators have reported a reduction in seed migration with the stranded sources.^(^ ^2^ ^,^ ^3^ ^)^ However, because the stranding process is performed by a company other than the seed vendor, some new problems have emerged. One of the problems is the additional turnaround time between the ordering and receiving of the seeds because of extra shipping processes between the seed vendor and the stranding company and the stranding process itself. In addition, a significant increase (approximately 60%–80%) occurs in the overall price of the seeds.

Favorable outcomes using stranded sources motivated the vendors to develop true linear source models that could resolve the problems associated with loose seed implants without creating additional problems.^(8)^ In this regard, RadioMed Corporation (Tyngsboro, MA) introduced the RadioCoil P103d sources for interstitial brachytherapy implants. Originally, RadioCoil sources were constructed in the form of a dense helix with a 0.35‐mm outer diameter. These sources were available in integral lengths ranging from 1.0 cm to 6.0 cm in 1‐cm increments. The helical structure not only provides better gripping within the implanted volume, but also enhances ultrasonic and radiographic visibility.^(9)^

Hartford et al.^(9)^ reported the first clinical application of RadioCoil P103d sources for the treatment of stages T1c – T2c prostate cancer. They noted excellent visualization of the RadioCoil P103d sources with fluoroscopy and ultrasound at the time of implant. In addition, post‐implant computed tomography scan confirmed the stability of the sources within the target volume and the improved dose homogeneity as compared with seed‐type implants.^(9)^

At our institution, the dosimetric characteristics of the original source design have been determined using both experimental and Monte Carlo simulation techniques.^(8)^ Evaluations of sources with active lengths ≤1 cm were performed using TG‐43U1 recommendations.^(10)^ However, because of the shortcomings of the TG‐43U1 protocol, dosimetric evaluation of the elongated source (that is, ≥1 cm) used the Cartesian coordinate system (“along and away” format). Although the tabulated data in the Cartesian coordinate system were useful for quick dose calculations, they could not be incorporated into current treatment planning systems, which are commonly based on TG‐43U1 parameters. Therefore, for clinical application of elongated sources, we introduced in a separate investigation two models as an interim solution for treatment planning with elongated sources.^(11)^ In those models, an elongated brachytherapy source was assumed to behave as a series of linear segmented sources (LSS) or as a series of point segmented sources (PSS). Both of these models are based on the TG‐43U1 parameters for 0.5‐cm or 1.0‐cm source segments. In that publication, the LSS model was demonstrated to be useful for replicating the Monte Carlo–simulated dose distribution of an elongated source (within ±4%) in the area bounded between the two ends of the active length of the source. However, outside the bounded area, up to 13% discrepancies were observed. Therefore, the need for TG‐43U1 parameters of elongated sources became apparent and vital with the introduction of a new RadioCoil P103d source design with a 0.8‐mm diameter. The following two problems (since resolved as described later in the present paper) were impeding achievement of this objective:

1. One of the unresolved problems was the lack of a definition and appropriate radial increment for the two‐dimensional (2D) anisotropy function of elongated sources in the TG‐43U1 recommendation. In a separate investigation, Awan et al.^(12)^ showed that implementation of the existing radial increment in the TG‐43U1 protocol for 2D anisotropy function may lead to a substantial error (≥30%) in the dose calculation for elongated brachytherapy sources. However, they recommended modifying the radial increments of 2D anisotropy functions to 0.2 cm, 0.5 cm, 1.0 cm, 1.5 cm, …, (L/2−0.2), L/2, (L/2+0.2), …, 4.5 cm, and 5 cm for elongated brachytherapy sources with the active length of L.
2. The lack of a National Institute of Standards and Technology (NIST) wide‐angle free‐air chamber (WAFAC) calibration system for sources longer than 1 cm was the second problem associated with implementing the TG‐43U1 protocol in dosimetric evaluation of elongated sources. Currently, the NIST WAFAC calibration system is capable of calibrating brachytherapy sources only ≤1.0 cm in length. In an independent investigation, we introduced an interim solution using a commercially available well‐type ionization chamber to calibrate elongated brachytherapy sources.^(13)^

The goal of the present project was to use the TG‐43U1 protocol with consideration of the above‐noted modifications to determine the dosimetric characteristics of the newly designed RadioCoil P103d brachytherapy sources. The determinations were performed using both experimental and Monte Carlo simulation techniques. In addition, dose profiles in an along‐and‐away format for a 4.0‐cm RadioCoil P103d source were determined for the purpose of comparison with previously published values.^(8)^

## II. MATERIALS AND METHODS

### A. The RadioCoil linear brachytherapy source

**Figure 1:**
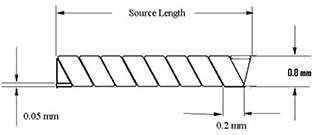
Schematic of the newly designed RadioCoil P103d brachytherapy source (courtesy of RadioMed Corporation, Tyngsboro, MA).

The new design of the RadioCoil P103d brachytherapy source was introduced for interstitial brachytherapy treatments. These sources are composed of a coiled ribbon in the form of a dense helix and are available in integral lengths ranging from 1.0 cm to 6.0 cm (with a tolerance of 0.5 mm). In addition to the clinically available active extended‐length sources, the vendor provided us with several 0.5‐cm sources for the purpose of comparison with other seed‐type sources. These sources are fabricated from a ribbon of high‐purity rhodium, which is bombarded with protons in a cyclotron to produce radioactive P103d. The palladium activity (approximately 1% by atomic composition) is uniformly distributed throughout the coil. The newly designed RadioCoil sources are 0.8 mm in diameter. Fig. 1 shows a schematic of the newly designed sources. The larger diameter eases source handling and also increases the activity per unit length. The coiled structure of the sources enhances their ultrasonic visibility and provides a better “grip” within the implanted tissue, which reduces the chance of source migration and embolization. The available activity of these sources can range from 0.1 mCi/cm to 13 mCi/cm per linear centimeter (0.13−16.8 U/cm).

### B. Dosimetry technique

Dosimetric characteristics of the newly designed RadioCoil P103d sources were determined experimentally and theoretically following the updated TG‐43 (TG‐43U1) protocol from the American Association of Physicists in Medicine (AAPM)^(10)^ and in accordance with the recommendations of Awan et al.^(12)^ and Meigooni et al.^(13)^ The subsections that follow describe the procedures for the experimental and Monte Carlo simulation techniques used in the investigations.

#### B.1 Experimental dosimetry technique

Dosimetric characteristics of the newly designed RadioCoil P103d sources with active lengths of 0.5 cm, 1.0 cm, and 4.0 cm were measured in Solid Water (Gammex RMI, Middleton, WI) phantom material. The measurements were performed using LiF thermoluminescent dosimeters (TLD‐100: Harshaw/Bicron, Solon, OH). The general experimental procedures and phantom designs used in the present investigation are similar to those described in previous publications.^(8,14–16)^ However, the subsections that follow describe specific procedures as required.

The dose rate constants of the 0.5‐cm, 1.0‐cm, and 4.0‐cm sources were measured using at least 3 separate sources from each active length. The first set of 0.5‐cm and 1.0‐cm sources were directly calibrated by NIST (NIST WAFAC 1999 calibration standard, corrected in 2000). The remaining sources were calibrated using a Capintech CRC‐7 well‐type ionization chamber (Capintech, London, ON) used as a secondary calibration system, following the procedures recommended in our previous publication.^(13)^ The measurements of the dose rate constants were repeated at least twice to verify the reproducibility of the data and to improve the statistical accuracy of the measurements. The final dose rate constant for each source length was extracted from the average of at least 30 TLD chips. Table 1 shows the error propagation for the experimental data in the investigations.

**Table 1:** Propagation of errors estimated for the experimental and Monte Carlo procedures used in the present investigations^a^

Component	Experimental uncertainty (%)
	Type A^b^		Type B^c^
Repetitive measurements	4.5		
TLD dose calibration (including LINAC calibration)			2.0
LiF energy correction			5.0
Measurement medium correction factor			3.0
Source/TLD positioning			4.0
Quadrature sum	4.5		7.3
Total uncertainty		8.6	
ADCL $S_{K}$ uncertainty		1.6	
Total combined uncertainty in Λ		8.7	

	Monte Carlo simulation	
	r=1.0 cm	r=1.0 cm
Statistics	0.9	1.2
Photoionization	d(1,θo)=0.66	d(5,θo)=3.4
Cross‐sections	2.3%	2.3%
Seed geometry	0.2	0.2
Source energy spectrum	d(1,θo)=0.1	d(5,θo)=0.1
Quadrature sum	1.14	3.6

a All values provided are for 2 σ.

b Statistical uncertainties.

c Systematic uncertainties.

The radial dose functions of the 0.5‐cm, 1.0‐cm, and 4.0‐cm sources were measured at radial distances ranging from 0.5 cm to 2.0 cm at 0.5‐cm increments using 1‐mm^3^ TLD cubes, and from 3.0 cm to 7.0 cm at 1.0‐cm increments using the 3.1×3.1×0.9‐mm TLD chips. The TLDs were placed at the transverse bisector plane of the sources. For each active length, the measurements were performed using at least 6 different sources, received in 3 separate shipments. The measurements were repeated at least twice to improve the statistical uncertainty of measurements (see Table 1). The final radial dose function at each radial distance was obtained by averaging the data from 40 different TLD values.

The 2D anisotropy functions of the above noted source lengths were measured in a custom‐designed Solid Water phantom by placing TLD cubes in circular arrangements around a horizontal source holder.^(^ ^14^ ^–^ ^16^ ^)^ A total of 23 TLD chips were placed at 1.0‐cm radial distances in 15‐degree angular increments from 0 degrees to 345 degrees for the sources with active lengths of 0.5 cm and 1.0 cm. Also, 36 TLD chips were placed in each concentric circle for radial distances of 2.0 cm, 3.0 cm, and 5.0 cm at 10‐degree angular increments from 0 degrees to 350 degrees for theses sources. For the 4.0‐cm sources, 2 TLDs at 0 degrees and 180 degrees for radial distances of 1.0 cm and 2.0 cm were skipped, because these points fall on the active length of the source. For the 2D radial dose function, results were extracted by averaging the anisotropy function in one quadrant. Each measurement was repeated at least twice to achieve better statistics. The final 2D anisotropy functions were obtained by averaging the data from at least 10 different TLD readouts.

In addition to the above noted TG‐43U1 parameterization of the sources, the dose distribution around a 4.0‐cm RadioCoil P103d source was measured in an along‐and‐away format. These measurements were performed using a specially designed and machined Sold Water phantom as described in our previous publication.^(8)^ This phantom material accommodates a 4.0‐cm source and a total of 104 TLD chips arranged in 8 rows parallel to the longitudinal axis of the source and 13 columns perpendicular to the longitudinal axis of the source. To be consistent with the relevant diagram in our previous publication,^(8)^ the distances between the rows and the longitudinal axis of the source are called “x” and the distances from each column to the transverse bisector of the source are called “z.” These TLDs were symmetrically distributed relative to the main axes of the source—that is, 4 rows on each side of the longitudinal axis of the source, 6 columns on each side of the transverse bisector, and 1 column on the transverse bisector. The spacing between the columns and rows was 0.5 cm, and the first row was 0.5 cm from the longitudinal axis of the source. These measurements were repeated 3 times to attain a statistical fluctuation less than ±3%.

#### B.2 Monte Carlo simulation technique

In the present investigations, we used MCNP5 Monte Carlo code(^12,17–19)^ to determine the dosimetric parameters of elongated RadioCoil P103d sources. The TG‐43U1 parameters were calculated in water and Solid Water phantom materials. In the simulations, F4 tally and an energy‐dependent mass‐energy absorption coefficient were used to obtain the absolute dose rates in the phantom.^(19)^ This code uses a default photon cross‐section library, p04, from ENDF/ B‐VI.^(20)^ The mass absorption coefficients of Hubbell and Seltzer^(21)^ distributed by NIST were used to obtain absorbed dose from energy flux. The photon energy cut‐off was set to 5 keV, which is consistent with the NIST WAFAC calibration standard.^(22)^

In the simulations, the coiled sources were modeled as rhodium cylindrical shells (50 μm thick) with uniformly distributed P103d activity throughout the wall. The sources were virtually placed at the center of a phantom consisting of a spherical volume of liquid water or Solid Water with a radius of 25 cm. Assuming a cylindrically symmetric source structure, circular annulus cells (diameter: 0.05 mm) around the longitudinal axis of the source were employed as the tally cells to score the eligible events. The dose rate constant and radial dose functions were determined by placing the tally cells at the transverse bisector of the source at radial distances ranging from 0.2 cm to 1.0 cm in 0.2‐cm radial increments and from 1.0 cm to 8.0 cm in 0.5‐cm radial increments. Tally cells had the same chemical composition as the phantom materials. Densities and the chemical composition of the Solid Water, liquid water, and air in these Monte Carlo simulations were obtained from the AAPM TG‐43U1 report.^(10)^ Photon emissions and their abundance for palladium were also obtained from the TG‐43U1 report.^(10)^

The 2D anisotropy functions of the new RadioCoil P103d sources were simulated in spherical (radius: 20.0 cm) liquid water and Solid Water phantom materials. These calculations were performed for radial distances of 0.2 cm, 0.5 cm, 1.0 cm, …, (L/2−0.2), L/2, (L/2+0.2), …, 4.5 cm, and 5.0 cm. For radial distances greater than L/2, anisotropy functions were calculated for angles ranging from 0 degrees to 90 degrees in 5‐degree increments. However, for radial distances of L/2 or less, the minimum angles were selected in such a way that tally points would not fall on the active length of the source.

Monte Carlo simulations for radial dose functions and 2D anisotropy functions were run up to $8\times 10^{7}$ starting particle histories. With that number of histories, the statistical uncertainties were found to be less than 2% for all tally points in liquid water and Solid Water. Table 1 presents the error propagation for the Monte Carlo simulation.

The air kerma strength, $S_{K}$, of each RadioCoil P103d source was calculated in a spherical (radius: 50.0 cm) air‐voided (vacuum) phantom. Tally points in those simulations were composed of dry air. The air kerma rates were calculated at radial distances ranging from 0.5 cm to 35 cm in 1.0‐cm radial increments. The air kerma strength of the source was calculated from the average of the simulated air kerma rates, multiplied by the square of the radial distances, in the range 20 – 30 cm. Monte Carlo simulations for $S_{K}$ were run for $2\times 10^{7}$ starting particle histories, which maintained statistical uncertainty of less than 0.3% for all the tally points. A detailed description of the Monte Carlo simulation for brachytherapy sources can be found in our previous publication.^(11)^

Dose profiles in the along‐and‐away format were simulated using the Monte Carlo code for a 4.0‐cm source using the same geometric setup described in the experimental procedures. Dose profiles were determined in both Solid Water and liquid water phantom materials. Monte Carlo–simulated values in Solid Water phantom material were compared with experimental data, and dose profiles in water were compared with previously published data.^(8)^

## III. RESULTS AND DISCUSSION

### A. Dose rate constant, Λ

Table 2 compares the calculated and measured dose rate constants in Solid Water phantom material of the newly designed RadioCoil P103d sources with active lengths of 0.5 cm, 1.0 cm, and 4.0 cm. The good agreement between the measured and Monte Carlo–simulated values indicates that the correct source and phantom geometries were used in the Monte Carlo simulations. Thus, the same geometric arrangements were used for Monte Carlo simulations of the dose rate constants for sources 1 – 6 cm in length in liquid water as shown in Table 3. In addition, the results indicate that the dose rate constants of the new source designs are within ±4% of the previously published data for sources with a diameter of 0.35 mm.

**Table 2:** Comparison of measured and Monte Carlo–simulated dose rate constants for RadioCoil P103d sources of 0.5‐cm, 1.0‐cm, and 4.0‐cm lengths and 0.8‐mm diameter, in Solid Water

Source length	Technique	Dose rate constant (%) $\Lambda \;({\text{cGh}}^{-1}U^{-1})$
0.5 cm	Monte Carlo	0.638±3
	TLD‐measured	0.643±7
1.0 cm	Monte Carlo	0.579±3
	TLD measured	0.588±7
4.0 cm	Monte Carlo	0.290±3
	TLD measured	0.294±7

**Table 3:** Comparison of Monte Carlo–simulated dose rate constants for a new model RadioCoil P103d source with previously published data in liquid water for sources with active lengths ranging from 0.5 cm to 6.0 cm

Active length	Source diameter		Difference
(cm)	0.35 mm	0.8 mm	(%)
0.5	0.650	0.663	1.96
1.0	0.597	0.602	0.83
2.0	0.457	0.473	3.38
3.0	0.350	0.363	3.58
4.0	0.278	0.286	2.80
5.0	0.230	0.235	2.13
6.0	—	0.201	—

### B. Radial dose function,$g_{L}(r)$

**Figure 2:**
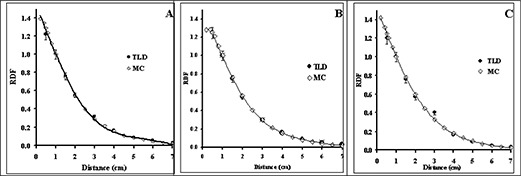
Comparison of measured [by thermoluminescent dosimetry (TLD)] and Monte Carlo–simulated (MC) radial dose functions (RDFs) for the RadioCoil P103d sources (RadioMed Corporation, Tyngsboro, MA) of (A) 0.5‐cm, (B) 1.0‐cm, and (C) 4.0‐cm lengths in Solid Water (Gammex RMI, Middleton, WI). The solid line in each graph is the 5th‐order polynomial fit to the Monte Carlo–simulated data.

**Figure 3:**
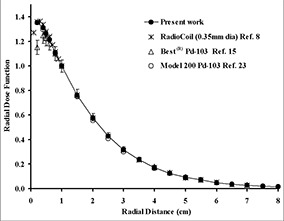
Comparison of the Monte Carlo–simulated radial dose functions of 0.5‐cm long RadioCoil P103d sources (0.8 mm and 0.35 mm in diameter) with other commercially available seed type sources.

Fig. 2 compares the TLD‐measured and Monte Carlo–simulated radial dose function of 0.5‐cm, 1.0‐cm, and 4.0‐cm sources in Solid Water phantom material. These results indicate good agreement (within 5%) between the measured and calculated data for each source length. The solid lines in this figure represent the 5th‐order polynomial fit to the Monte Carlo–simulated data. For clinical applications of the new source model, Monte Carlo–simulated radial dose functions for sources with active lengths of 1.0 – 6.0 cm were calculated in water (Table 4). In addition, for comparison with other seed‐type sources, Table 4 shows the radial dose function of the 0.5‐cm source in liquid water. In Fig. 3, the radial dose function of a 0.5‐cm RadioCoil source (diameter: 0.8 mm) is being compared with the published data for RadioCoil (diameter: 0.35 mm) and other commercially available P103d sources.^(^ ^8^ ^,^ ^15^ ^,^ ^23^ ^)^

**Table 4:** Monte Carlo–simulated radial dose function,$g_{L}(r)$, of RadioCoil P103d sources in liquid water phantom material

				Source length (cm)			
Distance (cm)	0.5	1.0	2.0	3.0	4.0	5.0	6.0
0.2	1.351	1.238	1.229	1.302	1.364	1.411	1.451
0.4	1.311	1.253	1.198	1.243	1.278	1.321	1.346
0.5	1.264	1.223	1.174	1.196	1.229	1.262	1.284
0.6	1.217	1.188	1.146	1.163	1.181	1.210	1.217
0.8	1.108	1.096	1.078	1.082	1.092	1.097	1.105
1.0	1.000	1.000	1.000	1.000	1.000	1.000	1.000
1.5	0.761	0.777	0.789	0.804	0.802	0.793	0.783
2.0	0.578	0.589	0.606	0.623	0.624	0.615	0.603
2.5	0.428	0.435	0.454	0.476	0.481	0.474	0.461
3.0	0.317	0.325	0.347	0.358	0.359	0.361	0.354
3.5	0.238	0.238	0.252	0.269	0.271	0.268	0.264
4.0	0.174	0.174	0.187	0.200	0.203	0.204	0.196
4.5	0.125	0.132	0.137	0.149	0.150	0.156	0.148
5.0	0.093	0.093	0.099	0.110	0.114	0.114	0.111
5.5	0.072	0.071	0.075	0.077	0.084	0.081	0.081
6.0	0.050	0.049	0.053	0.059	0.059	0.062	0.061
6.5	0.036	0.040	0.039	0.042	0.045	0.046	0.047
7.0	0.027	0.027	0.030	0.033	0.034	0.031	0.034
7.5	0.020	0.020	0.020	0.023	0.023	0.025	0.024
8.0	0.015	0.015	0.015	0.017	0.018	0.020	0.020

### C. 2D Anisotropy functions, F(r, θ)

**Figure 4:**
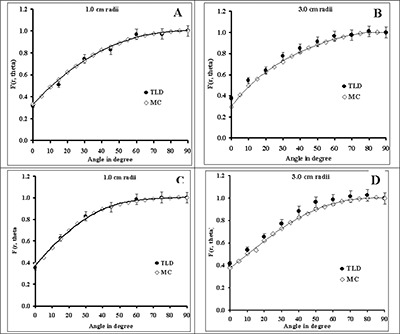
Comparison of the measured and Monte Carlo–simulated (MC) anisotropy functions of the RadioCoil P103d sources of (A,B) 0.5‐cm and (C,D) 1.0‐cm length, at radial distances of 1.0 cm and 3.0 cm.

**Figure 5:**
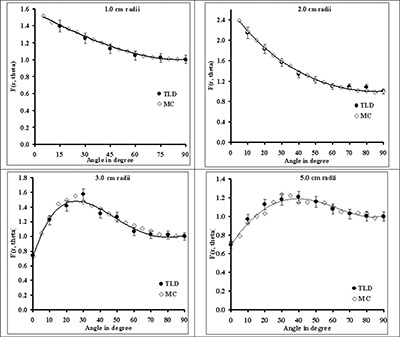
Comparison of the measured and Monte Carlo–simulated anisotropy functions of the RadioCoil P103d source of 4.0‐cm length, at radial distances of 1.0 cm, 2.0 cm, 3.0 cm, and 5.0 cm.

**Figure 6:**
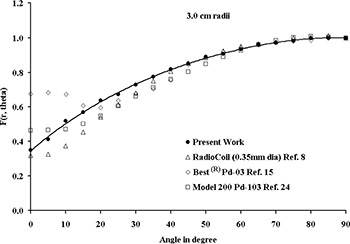
Comparison of Monte Carlo–simulated two‐dimensional anisotropy functions of RadioCoil P103d sources of 0.5‐cm length (0.35 mm and 0.8 mm in diameter) with the commercially available Best P103d and Pd‐103 Model 200 brachytherapy sources.

Figs. 4 and 5 compare the measured and Monte Carlo–simulated 2D anisotropy functions of, respectively, 0.5‐cm, 1.0‐cm, and 4.0‐cm RadioCoil P103d sources in Solid Water phantom material at various radial distances. The solid lines in these figures are the 4th‐order polynomial fit to the Monte Carlo–simulated data. The results show good agreement (within ±5%) between the measured and calculated data. Tables 5 – 10 present the Monte Carlo–simulated 2D anisotropy functions in water of RadioCoil P103d sources with active lengths of 1.0 – 6.0 cm. In addition, Table 11 shows the Monte Carlo–simulated 2D anisotropy function for the 0.5‐cm RadioCoil P103d source in water for comparison with conventional seed‐type sources. The 2D anisotropy function of the newly designed RadioCoil source with an active length of 0.5 cm is being compared with the published data for RadioCoil (diameter: 0.35 mm),^(8)^ Model 200,^(24)^ and Best P103d ^(15)^ sources (Fig. 6).

**Table 5:** Monte Carlo–simulated two‐dimensional anisotropy function of 1.0‐cm source in water as a function of radial distance

					Radial distance (cm)						
Angle (degrees)	0.2	0.5	1	1.5	2	2.5	3	3.5	4	4.5	5
0			0.374	0.317	0.331	0.329	0.366	0.321	0.307	0.274	0.292
5			0.437	0.406	0.407	0.406	0.415	0.419	0.432	0.419	0.435
10		0.995	0.530	0.483	0.479	0.479	0.492	0.505	0.502	0.500	0.507
15	1.057	0.977	0.613	0.561	0.548	0.539	0.547	0.553	0.562	0.542	0.549
20	1.003	0.977	0.690	0.635	0.616	0.611	0.616	0.614	0.624	0.609	0.611
25	0.989	0.982	0.752	0.696	0.682	0.677	0.679	0.686	0.685	0.670	0.677
30	0.987	0.986	0.807	0.757	0.741	0.730	0.727	0.724	0.721	0.714	0.715
35	0.989	0.990	0.848	0.804	0.787	0.778	0.778	0.784	0.772	0.770	0.764
40	0.987	0.990	0.889	0.848	0.830	0.817	0.821	0.821	0.816	0.801	0.811
45	0.992	0.994	0.915	0.880	0.868	0.856	0.855	0.854	0.855	0.844	0.852
50	0.995	0.995	0.936	0.910	0.899	0.896	0.896	0.897	0.895	0.868	0.873
55	0.996	0.997	0.953	0.932	0.926	0.921	0.920	0.922	0.922	0.916	0.908
60	0.993	0.998	0.963	0.951	0.947	0.946	0.943	0.950	0.941	0.934	0.936
65	0.998	0.999	0.980	0.971	0.968	0.962	0.966	0.974	0.968	0.955	0.949
70	0.997	0.996	0.987	0.977	0.977	0.973	0.975	0.981	0.985	0.982	0.967
75	0.996	0.999	0.996	0.989	0.989	0.988	0.990	0.986	0.987	0.982	0.975
80	0.998	0.999	0.997	0.993	0.995	0.993	0.998	1.005	0.995	0.999	0.999
85	0.995	0.997	1.000	0.999	1.004	1.000	1.007	1.011	1.001	0.988	0.991
90	1.000	1.000	1.000	1.000	1.000	1.000	1.000	1.000	1.000	1.000	1.000

**Table 6:** Monte Carlo–simulated two‐dimensional anisotropy function of a 2.0‐cm source in water as a function of radial distance

					Radial distance (cm)								
Angle (degrees)	0.2	0.5	0.8	1.0	1.2	1.5	2.0	2.5	3.0	3.5	4.0	4.5	5.0
0					0.718	0.483	0.413	0.389	0.336	0.400	0.353	0.353	0.347
5			1.278	1.278	0.804	0.595	0.507	0.484	0.481	0.478	0.475	0.459	0.466
10			1.235	1.215	0.933	0.737	0.626	0.573	0.561	0.548	0.559	0.520	0.533
15	1.116	1.069	1.212	1.189	1.023	0.849	0.722	0.667	0.645	0.619	0.624	0.600	0.607
20	1.045	1.068	1.187	1.167	1.062	0.928	0.804	0.741	0.718	0.704	0.683	0.681	0.660
25	1.022	1.065	1.163	1.148	1.079	0.985	0.874	0.822	0.782	0.761	0.753	0.734	0.725
30	1.013	1.056	1.134	1.134	1.083	1.011	0.924	0.873	0.843	0.820	0.809	0.790	0.786
35	1.009	1.051	1.111	1.113	1.076	1.028	0.961	0.907	0.888	0.864	0.849	0.839	0.824
40	1.004	1.048	1.095	1.089	1.073	1.039	0.984	0.944	0.918	0.901	0.884	0.873	0.875
45	1.004	1.038	1.074	1.076	1.061	1.039	0.998	0.963	0.945	0.925	0.926	0.917	0.902
50	1.000	1.030	1.056	1.063	1.049	1.034	1.010	0.981	0.968	0.953	0.945	0.935	0.919
55	1.005	1.021	1.045	1.045	1.041	1.032	1.010	0.989	0.985	0.972	0.977	0.953	0.950
60	1.005	1.018	1.031	1.034	1.028	1.026	1.009	0.993	0.992	0.982	0.978	0.961	0.965
65	1.007	1.017	1.022	1.023	1.020	1.018	1.007	0.996	0.994	0.987	0.991	0.971	0.982
70	1.001	1.011	1.015	1.013	1.009	1.014	1.010	1.002	0.997	0.990	1.000	0.976	0.990
75	1.003	1.004	1.009	1.007	1.004	1.010	1.006	0.999	0.995	0.999	1.000	0.982	0.986
80	1.000	1.005	1.003	1.002	1.001	1.002	1.006	1.000	1.001	1.000	1.012	0.995	0.999
85	1.000	1.001	1.001	0.996	1.001	1.002	1.005	0.994	1.005	0.997	1.005	1.011	0.995
90	1.000	1.000	1.000	1.000	1.000	1.000	1.000	1.000	1.000	1.000	1.000	1.000	1.000

**Table 7:** Monte Carlo–simulated two‐dimensional anisotropy function of a 3.0‐cm source in water as a function of radial distance

					Radial distance (cm)								
Angle (degrees)	0.2	0.5	1.0	1.3	1.5	1.7	2.0	2.5	3.0	3.5	4.0	4.5	5.0
0						0.961	0.651	0.534	0.527	0.420	0.490	0.418	0.454
5			1.378	1.658	1.663	1.152	0.834	0.685	0.630	0.616	0.589	0.571	0.575
10		1.128	1.351	1.591	1.561	1.322	1.046	0.842	0.760	0.709	0.703	0.654	0.652
15	1.141	1.103	1.332	1.509	1.497	1.375	1.177	0.979	0.883	0.831	0.775	0.748	0.739
20	1.069	1.096	1.300	1.432	1.430	1.366	1.236	1.074	0.979	0.910	0.859	0.855	0.822
25	1.045	1.089	1.271	1.365	1.371	1.326	1.246	1.115	1.038	0.978	0.929	0.911	0.889
30	1.032	1.081	1.231	1.306	1.319	1.297	1.242	1.150	1.079	1.024	0.981	0.953	0.932
35	1.026	1.064	1.194	1.250	1.264	1.251	1.220	1.148	1.103	1.037	1.008	0.980	0.973
40	1.018	1.061	1.163	1.206	1.211	1.213	1.189	1.146	1.105	1.081	1.043	1.020	1.006
45	1.017	1.046	1.127	1.169	1.169	1.172	1.154	1.126	1.099	1.066	1.032	1.020	1.024
50	1.013	1.039	1.101	1.131	1.135	1.133	1.125	1.105	1.089	1.060	1.030	1.030	1.019
55	1.009	1.029	1.078	1.098	1.104	1.103	1.101	1.088	1.070	1.058	1.037	1.032	1.033
60	1.002	1.020	1.059	1.071	1.074	1.077	1.069	1.069	1.057	1.043	1.030	1.010	1.001
65	1.004	1.017	1.041	1.048	1.048	1.049	1.051	1.043	1.049	1.029	1.023	1.005	1.026
70	1.008	1.010	1.026	1.030	1.032	1.031	1.027	1.032	1.031	1.015	1.006	1.006	1.014
75	1.005	1.004	1.019	1.018	1.014	1.016	1.021	1.016	1.014	1.016	1.015	1.011	0.998
80	1.004	1.007	1.003	1.008	1.002	1.006	1.003	1.005	1.008	1.004	0.991	1.000	1.004
85	1.003	1.001	1.003	1.004	1.002	0.998	0.999	1.004	1.007	1.008	0.990	0.996	1.012
90	1.000	1.000	1.000	1.000	1.000	1.000	1.000	1.000	1.000	1.000	1.000	1.000	1.000

**Table 8:** Monte Carlo–simulated two‐dimensional anisotropy function of a 4.0‐cm source in water as a function of radial distance

					Radial distance (cm)								
Angle (degrees)	0.2	0.5	1.0	1.5	1.8	2.0	2.2	2.5	3.0	3.5	4.0	4.5	5.0
0							1.333	0.909	0.721	0.623	0.963	0.554	0.733
5			1.435	1.820	2.216	2.201	1.652	1.209	0.964	0.881	1.139	0.793	0.742
10		1.177	1.383	1.769	2.055	2.032	1.821	1.484	1.190	1.055	1.368	0.898	0.889
15	1.174	1.146	1.350	1.696	1.889	1.897	1.806	1.598	1.342	1.199	1.496	1.015	0.981
20	1.092	1.126	1.318	1.608	1.747	1.761	1.727	1.608	1.411	1.315	1.616	1.130	1.073
25	1.063	1.116	1.278	1.523	1.622	1.647	1.619	1.569	1.442	1.336	1.642	1.182	1.138
30	1.038	1.100	1.243	1.434	1.515	1.546	1.537	1.491	1.413	1.341	1.624	1.207	1.199
35	1.034	1.089	1.206	1.361	1.419	1.443	1.447	1.425	1.383	1.332	1.553	1.208	1.208
40	1.037	1.079	1.170	1.295	1.335	1.353	1.361	1.357	1.332	1.300	1.493	1.216	1.199
45	1.033	1.060	1.143	1.235	1.272	1.284	1.291	1.282	1.271	1.251	1.400	1.200	1.182
50	1.019	1.049	1.108	1.182	1.211	1.221	1.226	1.221	1.220	1.209	1.329	1.150	1.171
55	1.012	1.040	1.087	1.140	1.157	1.168	1.166	1.171	1.172	1.160	1.255	1.108	1.121
60	1.008	1.033	1.060	1.100	1.115	1.125	1.129	1.122	1.127	1.128	1.200	1.099	1.092
65	1.012	1.027	1.043	1.070	1.078	1.079	1.082	1.082	1.083	1.084	1.143	1.074	1.082
70	1.005	1.018	1.026	1.043	1.045	1.049	1.053	1.048	1.057	1.060	1.087	1.036	1.046
75	1.010	1.014	1.016	1.024	1.027	1.025	1.026	1.027	1.035	1.034	1.043	1.028	1.029
80	1.003	1.008	1.004	1.011	1.010	1.009	1.005	1.007	1.008	1.021	1.033	1.010	1.022
85	1.001	1.004	0.994	1.003	1.001	0.999	1.003	0.996	1.001	1.005	1.015	0.998	1.014
90	1.000	1.000	1.000	1.000	1.000	1.000	1.000	1.000	1.000	1.000	1.000	1.000	1.000

**Table 9:** Monte Carlo–simulated two‐dimensional anisotropy function of 5.0‐cm source in water as a function of radial distance

					Radial distance (cm)								
Angle (degrees)	0.2	0.5	1.0	1.5	2.0	2.3	2.5	2.7	3.0	3.5	4.0	4.5	5.0
0								1.865	1.246	0.916	0.774	0.823	0.811
5			1.505	1.866	2.458	2.979	2.958	2.369	1.741	1.359	1.218	1.096	1.072
10		1.198	1.434	1.791	2.337	2.666	2.663	2.486	2.101	1.672	1.481	1.314	1.201
15	1.174	1.160	1.393	1.717	2.166	2.385	2.414	2.350	2.146	1.856	1.624	1.478	1.339
20	1.096	1.139	1.352	1.634	1.998	2.147	2.180	2.163	2.071	1.870	1.701	1.576	1.440
25	1.067	1.123	1.314	1.551	1.834	1.942	1.983	1.990	1.942	1.847	1.717	1.636	1.483
30	1.054	1.109	1.262	1.468	1.681	1.768	1.813	1.826	1.804	1.751	1.682	1.577	1.465
35	1.046	1.093	1.231	1.389	1.557	1.613	1.663	1.676	1.657	1.635	1.602	1.533	1.435
40	1.041	1.075	1.195	1.318	1.452	1.493	1.514	1.532	1.535	1.542	1.501	1.473	1.401
45	1.031	1.061	1.154	1.251	1.358	1.395	1.409	1.423	1.423	1.441	1.415	1.388	1.328
50	1.021	1.049	1.130	1.198	1.272	1.304	1.317	1.335	1.330	1.336	1.325	1.325	1.274
55	1.012	1.040	1.097	1.150	1.208	1.220	1.233	1.248	1.244	1.258	1.244	1.236	1.196
60	1.014	1.029	1.076	1.109	1.146	1.161	1.170	1.177	1.178	1.196	1.188	1.171	1.160
65	1.002	1.025	1.052	1.078	1.103	1.102	1.112	1.117	1.121	1.133	1.134	1.131	1.103
70	1.006	1.021	1.031	1.047	1.069	1.061	1.074	1.081	1.064	1.082	1.089	1.085	1.055
75	1.019	1.008	1.023	1.028	1.034	1.036	1.039	1.045	1.033	1.058	1.039	1.035	1.023
80	1.011	1.002	1.008	1.010	1.020	1.010	1.012	1.018	1.015	1.018	1.016	1.025	1.008
85	1.003	1.007	1.005	1.000	1.004	1.000	1.002	1.004	0.999	1.012	1.005	1.014	0.995
90	1.000	1.000	1.000	1.000	1.000	1.000	1.000	1.000	1.000	1.000	1.000	1.000	1.000

**Table 10:** Monte Carlo–simulated two‐dimensional anisotropy function of a 6.0‐cm source in water as a function of radial distance

					Radial distance (cm)								
Angle (degrees)	0.2	0.5	1.0	1.5	2.0	2.5	2.8	3.0	3.2	3.5	4.0	4.5	5.0
0									2.600	1.695	1.286	0.876	1.049
5			1.541	1.926	2.487	3.339	4.047	0.000	3.358	2.523	1.939	1.643	1.502
10		1.213	1.469	1.832	2.350	3.079	3.507	1.330	3.350	2.896	2.337	2.020	1.772
15	1.195	1.177	1.419	1.744	2.195	2.759	3.050	1.278	3.067	2.873	2.494	2.213	1.977
20	1.109	1.158	1.372	1.650	2.038	2.460	2.648	1.246	2.720	2.645	2.453	2.231	2.044
25	1.060	1.145	1.323	1.559	1.874	2.192	2.346	1.221	2.420	2.386	2.297	2.142	2.020
30	1.049	1.128	1.285	1.476	1.729	1.970	2.078	1.192	2.158	2.159	2.100	2.013	1.955
35	1.042	1.107	1.236	1.398	1.600	1.767	1.848	1.160	1.937	1.952	1.946	1.886	1.816
40	1.035	1.080	1.203	1.328	1.486	1.615	1.680	1.124	1.723	1.753	1.739	1.733	1.674
45	1.029	1.065	1.158	1.263	1.382	1.488	1.528	1.100	1.565	1.584	1.594	1.583	1.561
50	1.020	1.047	1.128	1.202	1.291	1.359	1.412	1.075	1.436	1.442	1.445	1.439	1.453
55	1.016	1.044	1.097	1.154	1.221	1.272	1.300	1.065	1.322	1.323	1.353	1.339	1.333
60	1.015	1.031	1.068	1.112	1.157	1.195	1.224	1.046	1.241	1.243	1.249	1.243	1.250
65	1.015	1.025	1.046	1.073	1.103	1.123	1.144	1.035	1.156	1.157	1.152	1.167	1.155
70	1.007	1.016	1.029	1.043	1.068	1.084	1.093	1.023	1.096	1.087	1.111	1.102	1.096
75	1.009	1.011	1.012	1.023	1.036	1.045	1.049	1.015	1.059	1.061	1.061	1.048	1.045
80	1.007	1.001	1.010	1.009	1.016	1.020	1.029	1.003	1.027	1.026	1.022	1.015	1.017
85	1.005	1.001	1.004	0.998	1.009	1.013	1.000	1.001	1.004	1.004	0.996	1.015	1.010
90	1.000	1.000	1.000	1.000	1.000	1.000	1.000	1.000	1.000	1.000	1.000	1.000	1.000

**Table 11:** Monte Carlo–simulated two‐dimensional anisotropy function of a 0.5‐cm source as a function of radial distance

			Radial distance (cm)			
Angle (degrees)	0.5	1.0	2.0	3.0	4.0	5.0
0		0.312	0.326	0.349	0.335	0.420
5		0.397	0.413	0.413	0.431	0.452
10	0.551	0.488	0.507	0.520	0.520	0.517
15	0.612	0.554	0.562	0.570	0.576	0.572
20	0.671	0.620	0.630	0.637	0.641	0.615
25	0.721	0.674	0.678	0.673	0.672	0.678
30	0.771	0.726	0.730	0.728	0.734	0.709
35	0.813	0.781	0.773	0.776	0.765	0.790
40	0.852	0.822	0.813	0.819	0.814	0.798
45	0.883	0.856	0.854	0.852	0.859	0.838
50	0.908	0.891	0.892	0.891	0.885	0.886
55	0.929	0.920	0.917	0.907	0.913	0.922
60	0.947	0.941	0.941	0.934	0.940	0.914
65	0.965	0.958	0.962	0.961	0.954	0.951
70	0.979	0.973	0.979	0.970	0.980	0.938
75	0.990	0.986	0.987	0.982	0.991	1.009
80	0.996	0.995	0.998	0.997	0.991	0.983
85	1.006	1.001	0.998	0.999	0.995	0.982
90	1.000	1.000	1.000	1.000	1.000	1.000

**Figure 7:**
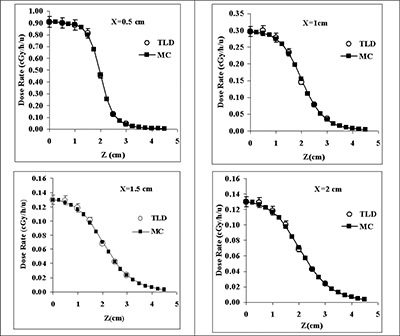
Comparison of the measured and Monte Carlo–simulated (MC) dose profile for a RadioCoil P103d source of 4.0‐cm length (0.8 mm in diameter), at radial distances of 0.5 cm, 1.0 cm, 1.5 cm, and 2.0 cm from the source axis.

Fig. 7 compares the Monte Carlo–simulated and experimentally measured dose profiles around a 4.0‐cm RadioCoil P103d source at radial distances of 0.5 cm, 1.0 cm, 1.5 cm, and 2.0 cm. These results present good agreement (within ±5%) between the two sets of dose profiles.

## IV. CONCLUSIONS

Experimental and theoretical techniques were used to evaluate the dosimetric characteristics of the newly designed RadioCoil P103d brachytherapy sources with a diameter of 0.8 mm and active lengths of 1.0 – 6.0 cm. The evaluations were based on TG‐43U1 recommendations,^(10)^ with consideration of the recently published recommendation by Awan et al.^(12)^ on determination of the 2D anisotropy function for elongated sources. In addition, in the absence of a NIST calibration system for elongated sources (active length: ≥1 cm), guidelines provided by Meigooni et al.^(13)^ were used to obtain $S_{K}$ for the elongated sources used in the experimental procedures.

The results of the investigations showed excellent agreement between the measured and calculated dose rate constants of various active source lengths in Solid Water (Table 2). In addition, the tabulated dose rate constant of the new source design was determined in liquid water for active lengths of 1.0 – 6.0 cm (Table 3). The dose rate constant for the 0.5‐cm source is also provided for comparison with other seed‐type sources. The results in Table 3 indicate that the dose rate constant of the sources decreases with increasing active length. Moreover, the dose rate constants of the 0.8‐mm diameter sources were found to be within 4% of the published values for the 0.35‐mm diameter sources. The agreement of the dose rate constants between the two source models can be attributed to identical wall thickness and identical distribution of activity within the wall.

The experimentally measured radial dose functions of the 0.8‐mm diameter RadioCoil sources were found to be in excellent agreement with the Monte Carlo–simulated data (Fig. 2). For clinical application of the new sources, radial dose functions were determined in liquid water using Monte Carlo–simulation techniques (Table 4). Good agreement was observed between the radial dose function of the newly designed (length: 0.5 cm; diameter: 0.8 mm) RadioCoil P103d source, the published data for the 0.35‐mm diameter RadioCoil source^(8)^, the Model 200^(23)^ and Best^(15)^ P103d seed‐type sources in water (Fig. 3).

The 2D anisotropy function of the newly designed elongated RadioCoil P103d sources with various active lengths were measured and calculated with Monte Carlo simulation techniques in Solid Water phantom material. The results showed good agreement (within ±5%) between the measured and calculated values (Figs. 4 and 5). Moreover, a comparison of those results indicated that the variation of 2D anisotropy function with radial distance is much larger when some of the selected radial distances are smaller than L/2 (compare Figs. 4 and 5). For clinical application, the Monte Carlo–simulated 2D anisotropy functions of the sources with active lengths of 1.0 – 6.0 cm were determined with Monte Carlo simulations (Tables 5 – 11). The results of these investigations showed good agreement between the 2D anisotropy function of the newly designed RadioCoil source and the published data for the 0.35‐mm diameter source^(8)^ and the Model 200^(24)^ and Best^(15)^ P103d seed‐type sources in water (Fig. 6).

**Figure 8:**
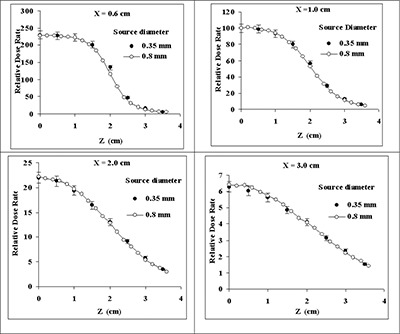
Comparison of Monte Carlo–simulated dose profiles along the longitudinal axis of RadioCoil P103d sources of 4.0‐cm length (0.35 mm and 0.8 mm in diameter). The dose profiles were calculated at distances of 0.6 cm, 1.0 cm, 1.5 cm, and 2.0 cm along the transverse bisector of the source.

Dose profiles of the newly designed elongated RadioCoil sources were experimentally determined in Solid Water, and the results were compared with the Monte Carlo data. Excellent agreement between these two sets of data was found (Fig. 7). In addition, Fig. 8 shows good agreement between the Monte Carlo–simulated dose profiles for the new design of the source (diameter: 0.8 mm) and the published data for the 0.35‐mm diameter source. The dose profiles from the two source models are in good agreement because both sources have the same wall thickness and a similar activity distribution within the wall. However, the larger diameter of the new source model results in larger activity per unit length and improved source handling.

In summary, our investigations yielded measured and Monte Carlo–simulated TG‐43U1– recommended dosimetric characteristics for the newly designed RadioCoil P103d sources. This information is available for treatment planning of brachytherapy implants with elongated RadioCoil P103d sources.

## ACKNOWLEDGMENT

The authors thank Ms. Jennifer Cole, Mr. David Pascoe, and Mr. James Eddy for their valuable suggestions and comments during the preparation of this manuscript. The work reported here was partially supported by U.S. Army Medical Research under DAMD 17‐02‐1‐0242 and by RadioMed Corp.
